# Visceral Leishmaniasis and Herpes Zoster as a Component of Syndrome of Immune Reconstitution Inflammatory Syndrome in an HIV-Positive Patient

**DOI:** 10.1155/2022/2784898

**Published:** 2022-03-14

**Authors:** Ermira Muço, Arta Karruli, Neada Hoxha, Alma Hoxhaj, Majlinda Kokici

**Affiliations:** ^1^Department of Infectious Diseases, Hospital University Center “Mother Theresa”, Tirana, Albania; ^2^Department of Precision Medicine, University of Campania “Luigi Vanvitelli”, Naples, Italy; ^3^Service of Laboratory, Hospital University Center “Mother Tereza”, Tirana, Albania

## Abstract

Immune reconstitution syndrome (IRIS) is a state of unusual hyperinflammatory response against latent infections which occurs after CD4 cell count improvement and as a consequence of immune response once highly active antiretroviral therapy for HIV is introduced. Leishmania parasites and varicella zoster virus (VZV) may be a manifestation of IRIS, but few data exist in literature in particular regarding Leishmania parasites. *Case Presentation*. A 47-year-old man was admitted to our hospital with fever. He was diagnosed with HIV infection and was a late presenter according to CD4+ count of 98 cells/mm^3^/9.5% and baseline illness (chronic diarrhea, weight loss, and oral candidiasis). The patient started highly active antiretroviral therapy (abacavir plus lamivudine plus efavirenz). Clinical symptoms improved and CD4+ increased to 22%, 374 cells/mm^3^. After 88 days, he presented with a 17-day history of high fever, sweat, fatigue, further weight loss, and lethargy. According to clinical image findings and hematochemical parameters, the patient was diagnosed with visceral leishmaniasis. He improved under treatment with liposomal amphotericin B. He presented again, 105 days after with disseminated herpes zoster infection. CD4+ count was 28.5%, 455 cell/mm^3^. The patient started treatment with acyclovir for 10 days. Four weeks later, he had no skin elements. At present, the patient continues HAART and is under regular monitoring. *Conclusions*. Early diagnosis of IRIS-associated diseases and treatment were fundamental in the patient's prognosis. Our patient presented with two different components of IRIS in two different time frames, confirming IRIS to be a broad-spectrum disease, heterogeneous and unique for each patient. A close monitoring during ART initiation, in particular in late presenters, is important in preventing IRIS. In case of IRIS development, a detailed investigation of rare associated diseases not only common ones is of great importance for the management and the prognosis of these patients.

## 1. Introduction

IRIS is a potential complication of highly active antiretroviral therapy (HAART). It was first reported in the 1990s. Following the initiation of HAART, however, there is an anticipated improvement in the immune-mediated inflammatory response. This unusual hyperinflammatory response is the trademark of IRIS. The prevalence of IRIS is likely to increase with the increasing use of HAART in human immunodeficiency virus (HIV)-infected patients. A few studies reported that up to 25–30% of HIV patients on antiretroviral therapy had IRIS [1, 2]. The clinical presentation depends on the underlying pathogen, organ/system involved, and the severity of the inflammatory response [3]. IRIS usually occurs within 6 months of initiation of antiretroviral therapy (ART) in patients with low CD4+ counts and can occur before any marked elevation in CD4+ count is achieved on HAART [4]. The spectrum of infections associated with IRIS is expanding and now includes a number of parasitic infections, which may be mediated by different immunopathological mechanisms. Cases of IRIS with visceral leishmaniasis (VL) have been reported [5]. In coexisting with HIV/AIDS, leishmaniasis surface in high prevalence countries presents frequently atypically, poses difficulties to be detected by routine diagnostic tests, and most often results in an unfavorable response to treatment, frequent relapses, and in premature deaths [6]. The coinfection Leishmania-HIV is frequent in the Mediterranean area [7] and more frequent in immunosuppressed patients (CD4 count <200 mm^3^) [7]. Mucocutaneous HZ accounts for 7–12% of the diseases related to HIV infection and can reactivate again when the subject's immunity improves from the administration of HAART. It usually occurs after 4 weeks from the initiation of HAART, and under these circumstances, the clinical symptoms and natural course of mucocutaneous HZ are similar to those in HIV-seropositive subjects who do not manifest IRIS [8].

## 2. Case Presentation

**Figure 1:**
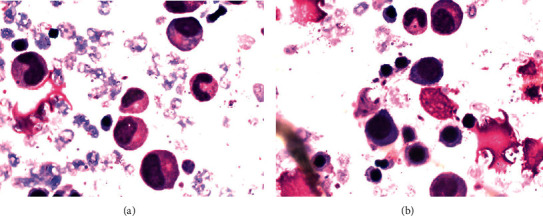
(a)-(b) Microscopic identification of Leishmania parasite in bone marrow aspirate.

**Figure 2:**
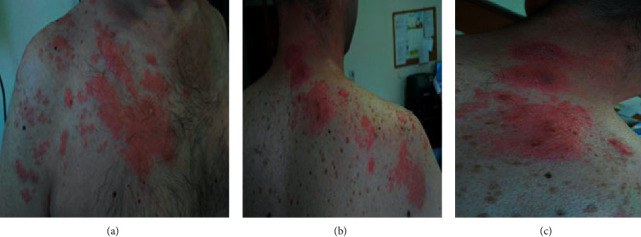
(a)–(c) Maculopapular lesions at different angles of herpes zoster. The three photos were taken on the same day. All stages of HZ can be seen: erythema, pustules, and crusts on the right thoracic region with dermatome C5, C6, C7, C8, and T1.

**Figure 3:**
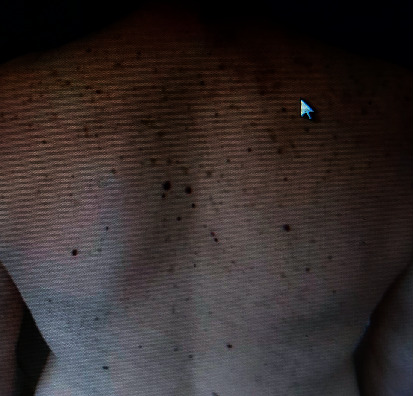
Resolved skin lesions of herpes zoster.

A 47-year-old male was diagnosed with HIV infection, stage C3, HIV-RNA was 2.4 × 10^4^, and CD4(+) count was 98 cells/mm^3^/9.5% at our infectious disease service of Mother Theresa Hospital, Tirana, Albania. The medical history of the patient was positive for oropharyngeal candidiasis, chronic diarrhea, and weight loss. The patient started ARV treatment, abacavir plus lamivudine plus efavirenz. He presented 88 days later with a 17-day history of high fever (39–40°C), sweat, fatigue, further weight loss, and lethargy. Physical examination revealed inguinal, cervical, and axillary lymphadenopathy and distended abdomen with hepatomegaly 3 cm and splenomegaly 4 cm under the costal margin. Hematochemical parameters showed pancytopenia: white blood cells 1.7 × 10^3^/mm³, red blood cells 2.66 × 10^6^ cells/mm³, hemoglobin 7.8 g/dl, hematocrit 22.3%, platelet count 1.31 × 10³ cells/mm³, lymphocyte count 51.8%, monocyte count 6.5%, and granulocyte count 41.7%. Other hematochemical parameters resulted as follows: C-reactive protein 117 mg/l, fibrinogen 554 mg/dl, the protein electrophoresis showed gamma globulin 36.1%, and albumin, 44.7%. Serological markers for *Toxoplasma gondii*, *Cytomegalovirus*, *Salmonella*, *Brucella*, hepatitis A and B viruses, and Epstein–Barr virus were negative. Autoimmune diseases were excluded. Bone marrow aspiration finally identified the presence of Leishmania (Figures 1(a) and 1(b)). IFAT and the rapid immunochromatography test-rK39 were positive. Ultrasonography examination revealed hepatosplenomegaly (liver 18 cm, spleen 19 × 10 cm). Investigation of the immune system showed CD4+ T lymphocyte count 22%, 374 cells, and CD8 T cells 61%. HIV-RNA was undetectable. The first cycle of liposomal amphotericin B (at a dose of 3–5 mg/kg/day) course was administered immediately for 5 days resulting in clinical and hematochemical parameters improvement (white blood cells 3 × 10^3^ cells/mm³, red blood cells 3.7 × 10^6^ cells/mm³, and platelet count 1.86 × 10^5^ cells/mm³). Combined amphotericin B and corticosteroid treatments were well-tolerated. Liposomal amphotericin B was administered again on days 10, 17, 24, 31, and 38. His general condition was both clinically and biologically stable. After 105 days under ARV medication, he presented to our hospital with maculopapular lesions that progressed into vesicles and then pustules and crusts on the right thoracic region with dermatomes C5, C6, C7, C8, and T1 (Figure 2). He was diagnosed with herpes zoster. The patient was treated with acyclovir for ten days. CD4 count was 28.5%, 455 cells. After four weeks, he had no elements in the skin, as shown in Figure 3. The patient did not develop postherpetic neuralgia. At present, the patient continues treatment with ART and is under regular monitoring. His life quality has improved significantly.

## 3. Discussion

Since the identification of the first HIV/AIDS case in Albania in 1993, 1430 HIV-positive persons have been diagnosed. 749 people receive ARV treatment. There have been publications about AIDS cases, but few data exist about IRIS; this complication is related to HAART. Immune reconstitution inflammatory syndrome (IRIS), also known as immune restoration disease, refers to a disease or pathogen-specific inflammatory response in HIV-infected patients that may be triggered after initiation or reinitiation of highly active antiretroviral therapy (HAART) or change to more active HAART therapy. The risk of IRIS is mainly associated with severe immunosuppression at the start of HAART [9, 10]. Our patient began HAART therapy at an advanced stage of immunodeficiency with HIV-RNA of 2.4 × 10^4^ and CD4(+) count of 98 cells/mm^3^, 9.5%. Males were more at risk of developing IRIS [11]. Also, the rate of HIV infection is higher in males compared to females [12]. The spectrum of infections now recognized as associated with IRIS is expanding and includes a number of parasitic infections, which may be mediated by different immunopathological mechanisms. Here, we included also visceral leishmaniasis [5]. Visceral leishmaniasis (VL) is a common opportunistic infection in HIV-positive patients from endemic countries but occurs rarely following antiretroviral treatment [13]. It is a vector-borne infection that is transmitted to humans through the bite of an infected sandfly which usually becomes infected from blood of infected animals in particular dogs. Clinical symptoms mostly include fever, weight loss, liver, and spleen enlargement which were found in our case as well. Albania is an endemic country for Leishmania [14]. In 2014, Badaro et al. noted that 34 cases of leishmaniasis as a manifestation of IRIS have been described worldwide [15]. The mean time between the onset of HAART and the occurrence of leishmaniasis IRIS-related manifestations was 6 days to 11 months [16, 17]. The interval between beginning of HAART and the onset of IRIS was 88 days in our case with VL. Mean onset of disease from ART initiation was 5 weeks (range 1–17 weeks), and no cases with varicella zoster occurred before 4 weeks of therapy [18, 19]. The interval between beginning of HAART and the onset of IRIS was 105 days in our case with VZ. IRIS is usually accompanied by an increase in CD4 cell count and/or a rapid decrease in viral load. CD4+ T lymphocyte count was 22%, 374 cells/mm^3^ when VL manifested, and CD4+ count was 28.5%, 455 cells/mm^3^ when VZ manifested. HIV-RNA was undetectable in our case. Significant increases in CD8 T cells are a risk factor for developing dermatomal zoster. We noticed in our patient an increase of CD8 T cells up to 61%. The pentavalent antimonials still remain the first line of therapy for coinfected cases. However, amphotericin B, especially the liposome formulation, the active component of AmBisome, was found to be relatively less toxic and more potent compared to pentamidine [6]. The cycle of liposomal amphotericin B course was administered immediately for 5 days which improved clinical and hematochemical parameters of our patient. Liposomal amphotericin B proved to be effective and safe in our case. No adverse event was observed. Combined amphotericin B and corticosteroid treatments were well-tolerated. Currently, new compounds are being studied such as curcumin, which showed great antileishmanial activity [20]. When the patient developed VZ, he was treated with oral acyclovir which also significantly improved the clinical situation.

## 4. Conclusion

Our patient was diagnosed as late presenter HIV, and since IRIS in HIV is associated with important immune system depletion at ART start, IRIS in our patient manifested not only with Herpes Zoster but also with visceral leishmaniasis, a less common IRIS-associated condition. Our patient presented with two different components of IRIS in two different time frames, confirming IRIS to be a broad-spectrum disease heterogeneous and unique for each patient. Early diagnosis and treatment were fundamental in the patient's prognosis. A close monitoring during ART initiation, in particular, in late presenters is important in preventing IRIS. In case of IRIS development, a detailed investigation of rare associated diseases not only common ones is of great importance for the management and the prognosis of these patients.

## Data Availability

No data were used to support this study.
